# Effect of immediate dentin sealing on the fracture strength of indirect posterior restorations:Systematic review and meta-analysis protocol.

**DOI:** 10.12688/f1000research.156876.2

**Published:** 2025-08-29

**Authors:** Yosra Gassara, Rim Kallala, Rihab Dakhli, Sarra Nasri, Nourhen Klach, Belhassen Harzallah

**Affiliations:** 1 Fixed prosthodontics, University of Monastir Faculty of Dental Medicine of Monastir, Monastir, Monastir, 5000, Tunisia

**Keywords:** Fracture Strength, Endocrown, Overlay, occlusal veneer, Indirect restoration, Inlay, Onlay, immediate dentin sealing

## Abstract

**Background:**

Immediate Dentin Sealing (IDS) is a technique that applies an adhesive layer immediately after tooth preparation, prior to the placement of indirect restorations. This method is gaining attention due to its potential to improve the bond strength and mechanical properties of restorations, particularly in the posterior region where restorations endure significant masticatory forces. The objective of this systematic review and meta-analysis is to evaluate the effect of IDS on the fracture strength of indirect posterior restorations.

**Methods:**

Following PRISMA guidelines, a comprehensive literature search was conducted in four databases: Web of Science, Scopus, Cochrane Library, and PubMed. Inclusion criteria focused on in vitro studies involving human teeth with indirect posterior restorations, published between 2014 and 2023. Studies using IDS were compared to those using conventional methods. The risk of bias was assessed using the Cochrane Collaboration’s tool, and a meta-analysis was performed with a random-effects model. The standard mean difference and 95% confidence intervals were calculated to compare fracture strength, with I ^2^ statistics assessing heterogeneity. Forest and funnel plots were employed to visualize results and publication bias, respectively.

**Discussion:**

The results indicate that IDS generally enhances the fracture strength of indirect posterior restorations, particularly with ceramic materials like lithium disilicate. However, the effectiveness of IDS varies depending on the material and restoration type. While some studies demonstrated improved fracture resistance with IDS, others showed minimal benefit, especially with composite restorations. Additionally, IDS improved marginal adaptation and internal fit but increased the risk of severe failures, such as root fractures.

**Systematic review registration:**

PROSPERO: CRD42024584545 (Registered on 08/09/2024).

## Introduction

Immediate Dentin Sealing (IDS) has emerged as a highly influential technique in the field of restorative dentistry, particularly in the context of indirect posterior restorations. ^1– 4^ This technique involves the application of an adhesive layer immediately after tooth preparation and before the final restoration is placed. ^5, 6^ The primary goal of IDS is to optimize the bond strength between the tooth and restoration, while simultaneously preserving the vitality of the tooth by creating a durable seal. In contrast to delayed dentin sealing, which occurs after temporization, IDS is performed in the initial stages of the restorative process, leading to enhanced adhesion and improved long-term outcomes. ^7^

The technique has gained significant attention for its potential to improve the mechanical properties of partial restorations, particularly those located in the posterior region of the mouth, where restorations are exposed to substantial masticatory forces. ^8– 11^ These forces make the posterior region especially vulnerable to mechanical failure, making it critical for restorations in this area to exhibit both high durability and fracture resistance. For these reasons, it is important to investigate how IDS impacts the long-term success of indirect posterior restorations, especially in terms of their ability to withstand the stresses of everyday function. ^8^

Despite its growing popularity, the effects of IDS on fracture strength remain a topic of debate. While several studies suggest that IDS enhances the fracture resistance of indirect restorations, discrepancies exist depending on factors such as the materials used, the specific restoration type, and the methods employed. Therefore, a systematic review and meta-analysis are necessary to consolidate the existing evidence and provide a clear understanding of IDS’s role in improving fracture strength. ^12, 13^

This systematic review and meta-analysis aim to assess the effect of IDS on the fracture strength of indirect posterior restorations, offering a thorough evaluation of current evidence.

## Protocol

### Methods

This systematic review will follow the Preferred Reporting Items for Systematic Review and Meta-Analysis Protocols (PRISMA-P) checklist. It will also comply with the Methodological Expectations of Cochrane Intervention Reviews and the Cochrane Handbook for Systematic Reviews of Interventions. The review protocol has been registered with the International Prospective Register of Systematic Reviews (PROSPERO) under registration number CRD42024584545, as of September 8, 2024.

### Objectives

**Primary objectives**

Specify the effect of immediate dentin sealing on the fracture strength of indirect posterior restorations.

**Secondary objectives**

The secondary objectives are to describe the benefits of IDS in posterior partial restorations, to present the mechanical properties of these restorations, and to specify the impact of IDS on their fracture resistance.

### Eligibility criteria

The PICOS framework (Participants, Intervention, Comparison, Outcome, Study Design) will be used to organize the eligibility criteria, which is intended to emphasize the key components of the research question.

**The research question for this systematic review is:**

The PICO question is: What is the effect of immediate dentin sealing (intervention) on the fracture strength (outcome) of indirect posterior restorations (population) compared to delayed dentin sealing (comparison)? ( Table 1)

**Table 1.** PICOS framework with inclusion and exclusion criteria.

Element	Inclusion criteria	Exclusion criteria
Population (P)	Human permanent posterior teeth restored with indirect restorations (inlays, onlays, overlays, occlusal veneers, endocrowns)	Anterior teeth; primary teeth; animal studies
Intervention (I)	Immediate dentin sealing (IDS) applied after tooth preparation (any adhesive protocol)	Delayed dentin sealing (DDS) only; provisional restorations without IDS
Comparison (C)	Conventional approach (DDS or no dentin sealing)	No control group
Outcome (O)	Primary: Fracture strength (in Newtons) of indirect posterior restorations; Secondary: Failure mode, reparability	Outcomes unrelated to fracture strength
Study Design (S)	In vitro comparative studies (RCTs or controlled laboratory studies) published in English (2014–2024)	Case reports, reviews, opinion papers, clinical trials without fracture strength outcomes

• **Types of participants:**

This systematic review will focus on teeth restored with indirect posterior restoration: Endocrown, Overlay, occlusal veneer, Inlay and Onlay.

• **Types of interventions:**

The intervention of interest is *immediate dentin sealing* (IDS). This technique involves sealing the dentin immediately after tooth preparation, before the indirect restoration is placed.

• **Types of outcomes:**

The primary outcome of interest is the *fracture strength* of the indirect posterior restorations.

• **Measures of effect:**

The main outcome, fracture strength, will be measured using either the *risk ratio (relative risk)* or *odds ratio*, depending on the type and availability of the data from the included studies. Meta-analysis will be performed if sufficient homogeneous data is available.

• **Study types:**

This review will include both *in vivo* and *in vitro* studies that assess the effect of immediate and delayed dentin sealing on fracture strength. Both randomized controlled trials (RCTs) and observational studies with a comparative design will be considered.

### Exclusion criteria

Studies will be excluded if they involve indirect anterior restorations, use animal models, or focus on interventions performed on temporary teeth. To ensure a neatly formatted protocol, the authors are encouraged to present information in paragraph form and reserve bullet points only for cases where they enhance clarity or readability.

### Search strategy

Two independent searchers, Y.G. and R.K. will search four different databases: Web of Science, Scopus, Cochrane library and PubMed.

They will perform a manual search and meticulously review the reference lists of the studies included in this review to identify eligible ones. Additionally, electronic searches in databases will conduct using a combination of mesh terms ( Table 2).

**Table 2.** The search strategy.

Database	Search strategy
**PubMed**	((“Fracture Strength” OR “Strength” OR “Force to fracture” OR “Fracture resistance”) AND (“Indirect posterior restorations” OR “Indirect restorations” OR “Endocrown” OR “Denture, Overlay” OR “occlusal veneer” OR “occlusal veneers” OR “Overlay” OR “Overlays” OR “Inlay” OR “Onlay”) AND immediate dentin sealing)
**Web of science**	(TS=(“Fracture Strength”) OR TS=(“Strength”) OR TS=(“Force to fracture”) OR TS=(“Fracture resistance”)) AND (TS=(“Indirect posterior restorations”) OR TS=(“Indirect restorations”) OR TS=(“Endocrown”) OR TS=(“Denture, Overlay”) OR TS=(“occlusal veneer”) OR TS=(“occlusal veneers”) OR TS=(“Overlay”) OR TS=(“Overlays”) OR TS=(“Inlay”) OR TS=(“Onlay”)) AND TS=(“immediate dentin sealing”)
**Scopus**	(TITLE-ABS(“Fracture Strength”) OR TITLE-ABS(“Strength”) OR TITLE-ABS(“Force to fracture”) OR TITLE-ABS(“Fracture resistance”)) AND (TITLE-ABS(“Indirect posterior restorations”) OR TITLE-ABS(“Indirect restorations”) OR TITLE-ABS(“Endocrown”) OR TITLE-ABS(“Denture, Overlay”) OR TITLE-ABS(“occlusal veneer”) OR TITLE-ABS(“occlusal veneers”) OR TITLE-ABS(“Overlay”) OR TITLE-ABS(“Overlays”) OR TITLE-ABS(“Inlay”) OR TITLE-ABS(“Onlay”)) AND TITLE-ABS(“immediate dentin sealing”)
**Cochrane**	"Fracture Strength” OR “Strength” OR “Force to Fracture” OR “Fracture Resistance” in Title Abstract Keyword AND ”Indirect posterior restorations” OR “Indirect restorations” OR “Endocrown” OR “Denture, Overlay” OR “occlusal veneer” OR “occlusal veneers” OR “Overlay” OR “Overlays” OR “Inlay” OR “Onlay” in Title Abstract Keyword AND ”immediate dentin sealing” in Title Abstract Keyword - (Word variations have been searched)

### Study selection (screening process)

To select studies, duplicates will be removed. Titles and abstracts will be reviewed by the two investigators, and studies that do not meet the inclusion criteria will be excluded.

### Assessment of methodological quality and risk of bias

The methodological quality of the included studies will be assessed using the QUIN tool (Quality Assessment Tool for In Vitro Studies), as all eligible studies are laboratory-based. This tool is specifically designed to evaluate in vitro studies, providing a structured assessment of methodological rigor.

### Data extraction

Data from the included articles will be collected by one author (Y.G.), while a second researcher (R.K.) will review all the extracted data for accuracy. The data to be collected will include the first author, year of publication, type of study, sample characteristics, IDS technique used, and fracture strength values. In addition to study characteristics, details of the adhesive/agent applied (etch-and-rinse, self-etch, universal, total-etch, protective liners, etc.) and the restorative material (lithium disilicate, polymer-infiltrated ceramic, composite resin, glass-ceramic, etc.) will be extracted. Information regarding the provisional phase, impression method, and storage conditions will also be recorded.

### Data analysis and synthesis

The meta-analysis will evaluate the fracture strength of indirect posterior restorations (inlays, overlays, occlusal veneers) by comparing conventional methods to the immediate dentin sealing (IDS) method. Studies that contrast IDS with conventional methods will be included. Data analysis will be performed using RevMan software (version 5.4). The standard mean difference with a 95% confidence interval will be applied, and the I ^2^ statistic will assess heterogeneity by measuring the percentage of variation across studies caused by true differences rather than chance. It helps determine if differences in study populations, methods, or interventions explain the variability.

Due to the variability in IDS protocols, restorative materials, and study setups, a random-effects model will primarily be used. For sensitivity analyses, results under a fixed-effects model will also be reported. Heterogeneity will be quantified using the I ^2^ statistic, with thresholds: I ^2^ <25%: low heterogeneity; 25–50%: moderate heterogeneity; >50%: substantial heterogeneity. Subgroup analyses will be performed based on the type of restorative material (ceramic, resin composite, polymer-infiltrated ceramic) and the type of adhesive agent (etch-and-rinse, self-etch, universal adhesives, additional protective liners). This approach will allow evaluation of whether IDS effectiveness differs across materials and bonding protocols.

To visually represent the results of the meta-analysis, forest plots will be generated. These plots will display the individual study outcomes along with the overall pooled effect, providing a clear illustration of the results and the degree of consistency among the studies. Additionally, funnel plots will be employed to evaluate the potential for publication bias. By plotting the effect sizes of the studies against their standard errors, funnel plots can help identify any asymmetry that may suggest selective reporting or other biases affecting the literature.

## Discussion

IDS generally enhances the fracture strength of indirect posterior restorations, especially with ceramic materials like lithium disilicate. ^12^ The effect of IDS, however, is material-dependent. ^14^^,^^15^ While it significantly enhances ceramic bonding, its influence is less pronounced or nonsignificant for multiphase resin composites. ^7^^,^^9^^,^^12^ IDS also improves microtensile bond strength, bonding durability, marginal adaptation, and internal fit in CAD/CAM ceramic restorations. ^4^^,^^6^^,^^11^ These findings align with previous in vitro studies, supporting IDS as a beneficial protocol for indirect restorations. ^7^^,^^12^^,^^13^ While many studies report improved fracture resistance with IDS, others show only modest benefits, particularly in composite restorations. ^12^ Additionally, IDS contributes to superior marginal adaptation and internal fit but may also elevate the risk of severe failures, such as root fractures. ^16, 17^

This review is limited by its exclusive reliance on in vitro studies, which may not fully replicate intraoral conditions. Differences in loading protocols, IDS techniques, and restorative materials contribute to heterogeneity and reduce generalizability. Sample sizes in included studies were relatively small, and some combined IDS with adjunctive treatments (e.g., proanthocyanidin), complicating interpretation. Long-term clinical trials are needed to validate laboratory findings.

This review serves as a valuable resource for clinicians, offering guidance when restoring posterior teeth with indirect restorations.

### Ethics and dissemination

Ethical approval is not required for this systematic review. The authors plan to present the results at relevant conferences and publish the findings in a peer-reviewed journal, following open science practices.

### Study status

The protocol of this systematic review was submitted to PROSPERO registry on 08th September, 2024 (CRD42024584545).

## Data Availability

No data are associated with this article. Figshare. PRISMA-P checklist for Effect of immediate dentin sealing on the fracture strength of indirect posterior restorations: Systematic review and meta-analysis Protocol. DOI: https://doi.org/10.6084/m9.figshare.27143277.v1 ^18^ The project contains the following dataset:
• PRISMA-P-checklist.docx Data are available under the terms of the Creative Commons Attribution 4.0 International license (CC-BY 4.0)
