# Supplementary material for: Effect of immediate dentin sealing on the fracture strength of indirect posterior restorations:Systematic review and meta-analysis protocol
Source: F1000Res. 2025 Aug 29;13:1234. Originally published 2024 Oct 15. [Version 2] doi: 10.12688/f1000research.156876.2 (PMC12449684; doi:10.12688/f1000research.156876.2)
Supplement: Peer review history

# Reviewer report: Reviewer response for version 2

**DOI:** 10.5256/f1000research.187134.r444543

**Published:** 2026-01-07

**Authors:** Álvaro Ferrando Cascales

**Affiliations:** 1 Universidad Católica de Murcia (UCAM), Murcia, Spain

- This systematic review and meta-analysis protocol is well structured and addresses a clinically relevant question regarding the effect of immediate dentin sealing (IDS) on the fracture strength of indirect posterior restorations. The rationale and objectives are clearly defined, and the proposed methodology follows established guidelines (PRISMA-P and Cochrane standards).
- The study design is appropriate for the research question, and the planned search strategy, data extraction, and statistical analysis are generally well described. However, some methodological aspects could be further clarified to enhance reproducibility, particularly regarding the interpretation of fracture outcomes across different restoration types and testing protocols.
- A specific point that would benefit from clarification concerns the discussion of catastrophic failures, such as roots fractures. While IDS has been associated with increased fracture strength, some studies report a higher incidence of non-repairable failures. This phenomenon appears to be mainly related to endocrown restorations, where stress transmission to pulpal and pericervical dentin is maximized, rather than to restorations with peripheral support such as overlays or onlays. Explicitly distinguishing between fracture modes and restoration designs would improve the biomechanical interpretation of the findings and prevent overgeneralization.

Overall, this protocol represents a valuable and timely contribution to the literature. Addressing the points above would further strengthen the clarity and clinical relevance of the final review.

Is the study design appropriate for the research question?

Yes

Is the rationale for, and objectives of, the study clearly described?

Yes

Are sufficient details of the methods provided to allow replication by others?

Partly

Are the datasets clearly presented in a useable and accessible format?

Not applicable

Reviewer Expertise:

Adhesive and restorative dentistry

I confirm that I have read this submission and believe that I have an appropriate level of expertise to confirm that it is of an acceptable scientific standard.

# Reviewer report: Reviewer response for version 2

**DOI:** 10.5256/f1000research.187134.r410704

**Published:** 2025-09-19

**Authors:** Cherif Adel Mohsen

**Affiliations:** 1 Faculty of Dentistry, Minia University, Minya, Egypt

Dear editorial board

 I accept this manuscript with only one comment which is reference number 1 is not fully cited.

 the topic entitled : effect of immediate dentin sealing on the fracture strength of indirect posterior restorations is nowadays.

 the manuscript is well written and answer many issues about the topic.

Is the study design appropriate for the research question?

Yes

Is the rationale for, and objectives of, the study clearly described?

Yes

Are sufficient details of the methods provided to allow replication by others?

Yes

Are the datasets clearly presented in a useable and accessible format?

Yes

Reviewer Expertise:

Fixed Prosthodontics

I confirm that I have read this submission and believe that I have an appropriate level of expertise to confirm that it is of an acceptable scientific standard.

# Reviewer report: Reviewer response for version 1

**DOI:** 10.5256/f1000research.172252.r401655

**Published:** 2025-08-11

**Authors:** Sanpreet Singh Sachdev

**Affiliations:** 1 Oral Pathology and Microbiology,, Bharati Vidyapeeth (Deemed to be University) Dental College and Hospital, Navi Munbai, India

PICOS criteria needs to be properly defined. Include a table of PICOS framework with specific inclusion and exclusion criteria. The current PICOS is very vague.

 Data extraction needs to have details of the agent applied and the restorative material used.

 In meta-analysis, specify the effects model and define heterogeneity values for those models - random and fixed.

 Include a section of subgroup analysis if the authors are including different restorative materials and adhesive agents, as these may differently influence the bonding process entirely.

 Limitations of the protocol need to be acknowledged and wherever possible, addressed.

 There are several spelling mistakes and grammatical errors in the manuscript such as 'objectif' or in the sentence stating the aim of the study. Similar errors are present sporadically in the manuscript. 

 The formatting of the manuscript is poor. The authors are suggested to write it in the form of paragraphs and use bullet points only when necessary for a neatly formatted protocol. The bullet points seem to be missing.

 The risk of bias tool mentioned may not be appropriate for in vitro studies. Since the authors are focusing on in vitro studies, they are suggested to use more suitable tools such as the QUIN tool.

Is the study design appropriate for the research question?

Yes

Is the rationale for, and objectives of, the study clearly described?

Partly

Are sufficient details of the methods provided to allow replication by others?

Partly

Are the datasets clearly presented in a useable and accessible format?

Partly

Reviewer Expertise:

Dentistry, Systematic Reviews

I confirm that I have read this submission and believe that I have an appropriate level of expertise to confirm that it is of an acceptable scientific standard, however I have significant reservations, as outlined above.

# Response

**Published:** 2025-08-23

**Authors:** Yosra Gassara

Dear Reviewer,

 We would like to thank you sincerely for your constructive feedback on our manuscript. Your comments have greatly contributed to improving the clarity, methodological rigor, and overall quality of our work. Below, we provide a detailed response to each point, along with the corresponding revisions made in the manuscript.

**Comment 1: PICOS criteria needs to be properly defined. Include a table of PICOS framework with specific inclusion and exclusion criteria. The current PICOS is very vague.**

**Response:** We appreciate this suggestion. The eligibility criteria have been revised to provide a precise PICOS definition. A detailed PICOS framework table has been added (Table 1), specifying participants, interventions, comparisons, outcomes, and study designs, along with both inclusion and exclusion criteria. This improves methodological transparency.

**Comment 2: Data extraction needs to have details of the agent applied and the restorative material used.**

**Response:** We fully agree. The data extraction plan has been expanded to include details of:

- The adhesive/agent applied (etch-and-rinse, self-etch, universal, protective liners, etc.),
- The restorative material used (lithium disilicate, polymer-infiltrated ceramic, composite resin, glass-ceramic, etc.).
  Information on the provisional phase, impression method, and storage conditions has also been included. These modifications ensure a comprehensive capture of all relevant variables influencing fracture strength.

**Comment 3: In meta-analysis, specify the effects model and define heterogeneity values for those models – random and fixed.**

**Response:** Thank you for pointing this out. We clarified that a **random-effects model** will be the primary model due to the anticipated variability in IDS protocols and restorative materials. For sensitivity analyses, results under a **fixed-effects model** will also be reported. Heterogeneity thresholds are now explicitly defined as:

- *I² < 25%*: low heterogeneity,
- *25–50%*: moderate heterogeneity,
- *>50%*: substantial heterogeneity.

**Comment 4: Include a section of subgroup analysis if the authors are including different restorative materials and adhesive agents, as these may differently influence the bonding process entirely.**

**Response:** We agree. A **subgroup analysis plan** has been added to stratify studies by:

- Type of restorative material (ceramic, resin composite, polymer-infiltrated ceramic),
- Type of adhesive agent (etch-and-rinse, self-etch, universal adhesives, protective liners).
  This will allow us to evaluate whether IDS effectiveness differs across materials and bonding protocols.

**Comment 5: Limitations of the protocol need to be acknowledged and wherever possible, addressed.**

**Response:** A **limitations section** has been added in the Discussion. It acknowledges:

- Restriction to in vitro studies, limiting direct clinical applicability,
- Variability in test setups, IDS techniques, and restorative materials,
- Potential for publication bias, despite efforts to minimize it.
  We also suggest that future well-designed clinical studies address these gaps.

**Comment 6: There are several spelling mistakes and grammatical errors in the manuscript such as “objectif” or in the sentence stating the aim of the study. Similar errors are present sporadically in the manuscript.**

**Response:** We apologize for these errors. The manuscript has undergone thorough proofreading and English language editing. Spelling (e.g., “objectif” → “objective”, “anlysis” → “analysis”)) and grammar have been corrected throughout.

**Comment 7: The formatting of the manuscript is poor. The authors are suggested to write it in the form of paragraphs and use bullet points only when necessary for a neatly formatted protocol. The bullet points seem to be missing.**

**Response:** Thank you for this feedback. The manuscript has been reformatted to improve readability. Paragraph structure has been refined, and bullet points are now used only when appropriate (e.g., inclusion/exclusion criteria, data extraction items). Tables have been harmonized for consistency with PRISMA guidelines.

**Comment 8: The risk of bias tool mentioned may not be appropriate for in vitro studies. Since the authors are focusing on in vitro studies, they are suggested to use more suitable tools such as the QUIN tool.**

**Response:** We appreciate this valuable suggestion. The **Cochrane risk of bias tool** has been replaced with the **QUIN tool (Quality Assessment Tool for In Vitro Studies)**, which is specifically designed for laboratory studies. This improves the validity and reliability of our methodological assessment.

 We are grateful for the reviewer’s constructive insights, which have allowed us to substantially strengthen the manuscript. We believe the revised version now addresses all concerns and meets the methodological standards expected.

 Sincerely

# Reviewer report: Reviewer response for version 1

**DOI:** 10.5256/f1000research.172252.r344743

**Published:** 2025-01-23

**Authors:** Dinesh Rokaya

**Affiliations:** 1 Ajman University, Ajman, United Arab Emirates

This is an interesting article done to evaluate the effect of IDS on the fracture strength of indirect posterior restorations.

 Abstract:

 The spelling of the Objective is a mistake. Please correct.

 The Introduction presents the background and Method explains in detail.

 PICOS framework (Participants, Intervention, Comparison, Outcome, Study Design is also clear.

 Does the IDS affect the bonding of the ceramic?

 More discussion is needed.

 Discuss with similar previous studies.

Is the study design appropriate for the research question?

Yes

Is the rationale for, and objectives of, the study clearly described?

Partly

Are sufficient details of the methods provided to allow replication by others?

Yes

Are the datasets clearly presented in a useable and accessible format?

Yes

Reviewer Expertise:

Dental Biomaterials, Prosthetic Dentistry

I confirm that I have read this submission and believe that I have an appropriate level of expertise to confirm that it is of an acceptable scientific standard.

# Response

**Published:** 2025-08-23

**Authors:** Yosra Gassara

**Reviewer Comment 1: The spelling of the Objective is a mistake. Please correct.**

**Response:** We thank the reviewer for pointing this out. The spelling error has been corrected from *“objectif”* to *“objective”*. The manuscript has also undergone a careful proofreading to ensure consistency in language and grammar throughout.

**Reviewer Comment 2: The Introduction presents the background and Method explains in detail. PICOS framework (Participants, Intervention, Comparison, Outcome, Study Design) is also clear.**

**Response:** We are grateful for this positive feedback. We are pleased that the revised Introduction, Methods, and PICOS framework are now considered clear and appropriately detailed.

**Reviewer Comment 3: Does the IDS affect the bonding of the ceramic?**

**Response:** Thank you for raising this important point. We have expanded the **Discussion section** to address the effect of Immediate Dentin Sealing (IDS) on ceramic bonding. Evidence suggests that IDS improves adhesive infiltration and reduces the risk of contamination, thereby enhancing the bond strength between resin cement and ceramic restorations. This effect is particularly relevant for lithium disilicate ceramics, where improved adhesion contributes to increased fracture strength and long-term restoration stability. A paragraph discussing this relationship, supported by recent literature, has been added.

**Reviewer Comment 4: More discussion is needed. Discuss with similar previous studies.**

**Response:** We agree that the Discussion required further depth. We have now expanded it by comparing our findings with previous systematic reviews and experimental studies that investigated IDS protocols and their effect on indirect restorations. These comparisons highlight consistencies (e.g., improved fracture strength with IDS) as well as discrepancies (e.g., variations in results depending on adhesive system or restorative material). This addition provides stronger context and situates our findings within the broader body of evidence.
